# Supplementary material for: Patterns of Intron Gain and Loss in Fungi
Source: PLoS Biol. 2004 Nov 30;2(12):e422. doi: 10.1371/journal.pbio.0020422 (PMC532390; doi:10.1371/journal.pbio.0020422)
Supplement: Table S1 — Also available at http://genes.mit.edu/NielsenEtAl/. (4.3 MB ZIP). [file pbio.0020422.st001.zip › NielsenEtAl/html/1442.html]

AN3627.1.NCU06265.1.MG05250.1.FG02585.1


```
 CLUSTAL W (1.82) Multiple Sequence Alignments - Introns Inserted


Sequence 1: NCU06265.1	887 aa
Sequence 2: FG02585.1	904 aa
Sequence 3: MG05250.1	839 aa
Sequence 4: AN3627.1	892 aa
Alignment Length: 945 aa
Number Identitical Residues: 288 aa
Alignment Score (without introns) 16862


MG05250.1 	------MSSVNKPVDVKQKDADIARKLQVYGIISAFQAGKIPS~NDQIDIALNSFLQTKA
NCU06265.1	MATVNGKANVNRPVDSKQKEADVNRKLQFYGIASAFQCGKVPS0NDQIDVALNSFLQSKA
FG02585.1 	MSTG---ANVNKPTNVQTKEADVNRKLQIYGIISAFQNGKVPS0NDQIDIALNSFLASRA
AN3627.1  	----MPSRAVNKPVDPKQRDKDINNKLQLFGIYHAFKNGKLPT0NKQCDVAINSALAHKA
          	         **:*.: : :: *: .***.:**  **: **:*: *.* *:*:** *  :*

MG05250.1 	LSKPSDKLSPEGQALVADFRDVVKKAQHLVLSKNEGNLLQDFIWQTTHFDPKSVQSTQAP
NCU06265.1	LANPSKKLSPEGQQLVADFREVVKEAKNLLLSKNEGNLLQDFIWQTQQFNPKAVSLPGAP
FG02585.1 	LSGNHEKLSTEGQQLVKDAANAIKQAKYLLLSKNEGNLIQDFIYQTTQFDPKSVNTPNAP
AN3627.1  	LTNPSKELSEEGRALVQDLRIIIEEAKKLILSKNDGQLLQEFIYDAQHISTEDVQKPNVA
          	*:   .:** **: ** *    :::*: *:****:*:*:*:**::: ::..: *. . ..

MG05250.1 	VDKDTARAHGNRAAEGLRTLGTLIITNGQFRKLL1KDASILLRDMAGDAATKAASNVKPS
NCU06265.1	VDKEQAQQHGNQALEGLRTLGTLIITNGQFRKLL1KDATVLLRDIAGDAATNAAARVRPS
FG02585.1 	VSKDAAKQDGDQALEGLRTLGQLLITNGQFRKLL~SDATVLFRDMAGDAATNAAARVRPS
AN3627.1  	GN-DTAEQDAARAREGLKTLGTLMITNGEFRKLL1NDAMIIARDIAGDASQKAADSVRPA
          	 . : *. .. :* ***:*** *:****:***** .** :: **:****: :**  *:*:

MG05250.1 	QDALSQIDRPADDNVWHDAPNLSKDNIRKQVQGAYKNN-KTNG~QAPATTQVGQIAP---
NCU06265.1	QEDLQQLDAPAPDNTWHEKPDFSRDQAKSKFSNIYKGNPKEDA~KHVLGEATSAAHP---
FG02585.1 	QEQLQQLDEPAQDNVWHEAPDFSKENFKKQAKGIYSGNPKQDA~KDIAAAGVNSAAPGQQ
AN3627.1  	EHELSQVDQPAEDNVWHEKPDLSGQ--KEQLKSRFSKN-KDKA0QQEASDVANTGAQ---
          	:. *.*:* ** **.**: *::* :  :.: .. :. * * .. :      ..       

MG05250.1 	--------GQTSP----VQSTAVQP-----------------------TTAGGNTQEPPP
NCU06265.1	-------DGSADP--RDLANTAAH---------DGRTGASSGIDTEKGLSSAANTAQRKL
FG02585.1 	QPLQQDQQGNLQPSEQEIHNQGAHPQTASGAPTDAARDAARQADPQAGKSAAKQVAEQKL
AN3627.1  	--------AATGP-----SGDSVDP------------------------YSGVNASLQHA
          	        .   *      . ....                         :. :.     

MG05250.1 	EVQLTAEAQQAAKQKGVEYRARTKRYLEQKVPKDRREATVWRLK~KMLIECQQHPEY~QT
NCU06265.1	DQNLDDETKEKAKKKKEEYKARTREYFKKKVPQERREQTIWRLK0KMIVEIQQHPDY~HR
FG02585.1 	DNKVDPETKENILQRNEEYRRKAHDYFNKKMPQERKDQTIWRLK~KMILECQQHEDY~SQ
AN3627.1  	QSKVPEDQKAEANKRSQEYKDRTKNYLSGKFPQERRDQTIYRLK~KMILEIQGHRDY1QQ
          	: ::  : :    ::  **: ::: *:. *.*::*:: *::*** **::* * * :*   

MG05250.1 	AIHTLLDLASEYGYHAKSLGMDSSEAAKDARASN-LGQAERDLK~TLIERFANGTSTDDL
NCU06265.1	AITTLLNIAEEYGSHANRLAQGGTSTVKDTRNA--LAQAEGDLK~TLIERFANGTSTDDL
FG02585.1 	AIQTLLRLAETYSRHGRTVGQGSTETAKGARGG--FGAAERDLR~TIIERFANGTSTEDL
AN3627.1  	AIETLLDLAEKYGGHGRHIAQQGTGSVQGTRGQSKVQTMEKNLR0TLIERFANSTSMDDF
          	** *** :*. *. *.. :.  .: :.:.:*  ...   * :*: *:******.** :*:

MG05250.1 	WNAIGQIYRDADHDPELRNWLKRCNRYIR~RCLEEQGYVIDESSTEEWDQLYDEGAYLLR
NCU06265.1	WASIKTIYEDADKDPELKNWFKAMNSFIR~RCLQEEGYILDDASNEEWNRLYDQGNYLLR
FG02585.1 	WEAIGQIYRDADNDRELKDWFKAMDSYIR~RCLLQQGYILEDQSTREWDQLYDQGRYLLR
AN3627.1  	FDSLENIYRDADKDPELKGWFKNTNQFIR2KTLQQQGYILQDDWNRQYNDLAEHGRFLLR
          	: ::  **.***:* **:.*:*  : :** : * ::**::::  ..::: * :.* :***

MG05250.1 	DKYKIHVDRILDEVKFLFSEFDKDPMNRAFREAIMKLFNDLGNDENGKPVFKKHLVKDLT
NCU06265.1	NKYRAHTDRVIDELKFLADQFDHDPQNKSFAASLNKLFTDLGNDENGKPTFKPHLVKDLT
FG02585.1 	NKYRGHTDRIVDEIKFLADQFDQDPQNHAFAETLTKLFKDLGNDQDGKPVFKPHLLKDLR
AN3627.1  	DRYRDHTNRVLDEIKFLGDQFNQDRQNRAFGDAVEKLFIDLGRDENGNPKFKKHLVKDIT
          	::*: *.:*::**:*** .:*::*  *::*  :: *** ***.*::*:* ** **:**: 

MG05250.1 	EVIVPATLENIAYVPIPRIEYQDSQIDFVVENLVLESDNFTPNVMEIDSSNYMRW0GRKT
NCU06265.1	DVVIPAMFENVAYIPVPRIEYSDHQIDAVIENLVLESDNFMPNVLEVASENYLRF~GRKK
FG02585.1 	DVIIPAALENIAYIPIPRIEYTDSQFDAIIENLVLESDNFAPNVLEVSSEHYFRW~GRKK
AN3627.1  	DVILPAIFENVRYVPIPRIEVQDAMADVVIENLVIESDNLMPNVVEFGSDNYYRW~GRKK
          	:*::** :**: *:*:****  *   * ::****:****: ***:*. *.:* *: ***.

MG05250.1 	VKSSRKHSGEIKVAGVQMDLRDVSYYVKRKKGFPSLTDVGLMDVFMGGNGFSFKIKVSTP
NCU06265.1	IANKKNMSVDVKVAGIQMDLRDVSYYIKRKQGFPSLSDTGIANILLEGDGFTFRMKMSSA
FG02585.1 	IANKNHQTMEVKVAGIQMDLRDVSFHIKRKQGFPSLTDTGVADIVLPGDGFSFKMKVSTA
AN3627.1  	ISSKRDNKIMISVSGIQADLRDVSFYINKKQGFPSITDTGVMDIFLGGEGFSFKIAASTA
          	: .... .  :.*:*:* ******:::::*:****::*.*: ::.: *:**:*::  *:.

MG05250.1 	DKKDAQNFFKVDKVDVEVNNLSIKIHQSNHKFLFSIAKPIMLKVMRPAIEKALGKVIKDR
NCU06265.1	DAKDRQNFFKIDKVDVDVKQFRIKLIKSNHKLLFNLFKPIMLKVLRPGLQKALEKAIRDQ
FG02585.1 	EKKDRQNYFKVEKVDVDFAKIQIKVKKSNHKLLFSILKPIMLKVIRAPLQKAVEKAIKDQ
AN3627.1  	QKQDRQNFVKLDKVSVKLDDIDIKLKKSKHKVLFTLFKPLLFRTVRPALQKALEKQVRDV
          	: :* **:.*::**.*.. .: **: :*:**.**.: **::::.:*. ::**: * ::* 

MG05250.1 	FNEFDQILFQLKQEADKALEEAQSDPENVPNIYSRYTSAIQRRAMATKKKTEEAVA----
NCU06265.1	ATRLDSILYEIKQEADRALEEAREDPEKTPNIYKRYVTAAQKKVLQGKQKAEGAVA----
FG02585.1 	CNKLDQLCYQVKQEAERATAEAKNNPENAPNIYNRYYTAAQKQFTQKKQKAQEVAS----
AN3627.1  	FEKADAFAYDVHQEAQRVKEHVKENPQDAPNIYNRYLNSFRAKLEEGRRKAQEASQQAAQ
          	  . * : ::::***::.  ..:.:*:..****.** .: : :    ::*:: .  .::.

MG05250.1 	-DKKVNYAVTTQDSIFPDIKLPGPANGSALSTRAAEFRELATKGSAWESPVFSVGEASKS
NCU06265.1	-DKKINYAVTKEDSIFPHISLPG-----GISSKATEYKELARKGEKWESPVFSIGSAGKS
FG02585.1 	-DKKANIAMTKEDSIFPNIDLPG-----GISTKATEYKELARKGDKWESPIFLVGSAKKS
AN3627.1  	RDTKAQTTTTLKGSLFPDIKLPG-----GITTKATEYEELARKGERWESPIFSIGSASES
          	 *.* : : * :.*:**.*.***     .::::*:*:.*** **. ****:* :*.* :*

MG05250.1 	SDVPAAPQITRKVHNTGNGN---------GVGAGQG-MVGSGPGMVNSGPGMVGGQP---
NCU06265.1	HDIPPAPRVVRKPHSTTGTG---TNG--AAHGTGNG-HALHGGPGMNVGNGSLNVGNDGL
FG02585.1 	TDIPAAPKIQRKAHPVSNLGPKETNADYDFYPAGNG-HQHTGNGAMN-GNGYHVGHGDGL
AN3627.1  	SDIPKPEQITRKPHNTAESKLRDRQSNGAATGGAAGGLAAAPRGPVTNGGALATGRATDG
          	 *:* . :: ** * .      . ...      . *.        :. * .       . 

MG05250.1 	--------------------------QLTNGGVPMYA----------------
NCU06265.1	N-LPGANYLPGDPAVSGF-------GTKNVGSAPNGRVTVGL-----------
FG02585.1 	NGGVHSKHLPGN--------------GVNNGQVPMAGQTTNIV----------
AN3627.1  	GPATAAPTLTNGSTAVTNGHAKHYDSLLSNGADGKLAQDISQISGTGFNPQTA
          	.    :   ....:    . :.  ..  . *         .  :.:. ...::
```
